# Comment on ‘Moral injury in clinical and academic medicine—it is time to act’: the role of stakes-dependent invisibility (*BJA Open* 2026; 17: 100525)

**DOI:** 10.1016/j.bjao.2026.100568

**Published:** 2026-05-28

**Authors:** Tumay Uludag Yanaral

**Affiliations:** Department of Anaesthesiology and Reanimation, Istanbul Medipol University Hospital, Istanbul, Turkey

**Keywords:** anaesthesiology, burnout, correspondence, effort-reward imbalance, moral injury, organisational justice, professional invisibility, surgical complexity

Editor

von Ungern-Sternberg and colleagues^1^ make a strong case for reframing clinician distress as moral injury rather than burnout, locating the cause in broken systems rather than broken individuals. The purpose of this letter is to add a mechanism that has not been named, though most anaesthesiologists will recognise it immediately: *stakes-dependent invisibility.*

Stakes-dependent invisibility in medicine refers to the phenomenon whereby healthcare providers, patients, symptoms, or harms remain unseen, disregarded, or unrecognised within the healthcare system, owing to power hierarchies, high-stakes clinical environments, or diagnostic limitations that permit such oversight.^2^

Anaesthesiologists’ contributions are under-recognised across surgical settings. Surveys spanning multiple countries and nearly two decades report that between 25% and 57% of patients do not know what their anaesthesiologist does,^3^^,^^4^ and fewer than 8% report experiencing meaningful shared decision-making with their anaesthesia clinician.^5^ What has not been examined is the influence of medical and surgical complexity and acuity on stakes-dependent invisibility. Notably, existing patient awareness studies have been conducted in routine surgical cohorts, and not in high-acuity operative settings such as organ transplantation, cardiac surgery, or major trauma.

In a routine hernia repair, the anaesthesiologist’s contribution is standardised, and the recognition deficit, while real, is modest at best. In organ transplantation or major trauma, the anaesthesiologist independently manages bleeding, coagulopathy, and haemodynamic collapse, often for hours. Systematic evidence on attribution patterns across surgical complexity tiers is limited, but illustrative cases are instructive. Examples include widely reported accounts of one prominent liver transplant case of the past two decades, which consistently name the surgeon while the anaesthesiologist remains unidentified.^6^^,^^7^ Whether this reflects broader patterns driven by cognitive salience, narrative convention, or institutional culture is a question that merits systematic examination beyond individual cases.

Three interdependent mechanisms may drive stakes-dependent invisibility for anaesthesiologists. First, *outcome salience bias*: anaesthetic value is defined by what does not happen—no cardiac arrest, no haemorrhagic crisis, no failed airway. The harder we work to prevent catastrophe, the more seamless the outcome appears. Second, the *surgeon-as-protagonist narrative*: perioperative communication and media reporting tend to frame the operating surgeon as the singular agent responsible for patient survival, marginalising other healthcare team contributions regardless of their clinical magnitude. Third, *structural invisibility*: anaesthesia care is delivered in spatial and temporal proximity to surgery but rendered institutionally separate, with limited formal mechanisms for attribution. While each mechanism matters less in routine practice, together, they may obliterate recognition of an extraordinary contribution in high-acuity surgery.

How does *stakes-dependent invisibility* relate to moral injury? The authors delineate circumstances in which clinicians recognize the ethically or clinically appropriate course of action yet are constrained from pursuing it.^1^ Production pressure, surgeon-related distracting behaviours, and consent dilemmas exemplify this pattern.^8^^,^^9^ The phenomenon described here overlaps with established occupational constructs. The effort-reward imbalance theory identifies inadequate recognition as a pathway to occupational ill-health^10^ and organisational justice frameworks similarly describe how institutional failure to acknowledge contribution generates distress.^11^ Stakes-dependent invisibility operates differently from the classic moral injury mechanisms. While the anaesthesiologist delivers optimal care, no ethical compromise occurs; however, the system simply does not recognise it. The moral distress is not “I could not do what was right.” It is “I did everything right, and it did not seem to matter to anyone.” This meets VanderWeele and colleagues’ definition of moral injury: persistent distress that disrupts one’s sense of the goodness of institutions.^12^

Several interventions, operating at different levels, could help address stakes-dependent invisibility. At the clinical encounter level, structured perioperative debriefs with patients and families before and after high-complexity cases could make the anaesthesiologist’s role more visible, as evidence shows that systematic self-introduction and interactive perioperative communication significantly improve patient understanding of the anaesthesiologist’s clinical role.^13^ At the institutional level, communication policies that mandate multidisciplinary attribution in internal outcome reports and external press releases, naming the surgeon and the anaesthesiologist, would appropriately give credit to all. At the professional and societal level, perioperative care models that position the anaesthesiologist as a visible partner throughout the surgical journey—before, during, and after the procedure—can reshape how the specialty is understood by patients, families, and administrators. Professional bodies are well placed to promote explicit attribution standards in their guidelines and position statements. This stakes-dependent invisibility hypothesis is testable: a prospective multicentre study comparing the contribution–recognition gap across surgical complexity tiers could determine whether it exists and if the acuity gradient is real. Until then, we are left with a troubling question that many anaesthesiologists have likely faced in silence: if a patient survives because of what you did, but no one ever recognizes the care you provided, how long before that silence becomes a moral injury?

## Declaration of generative AI and AI-assisted technologies in the writing process

During the preparation of this work, the author used ChatGPT (OpenAI) in order to assist with manuscript drafting and editing. After using this tool, the author reviewed and edited the content as needed and takes full responsibility for the content of the publication.

## Declaration of interests

The author declares no conflicts of interest.
